# Chronic neuropathic pain: EEG data in eyes open and eyes closed with painDETECT and brief pain inventory reports

**DOI:** 10.1016/j.dib.2023.109060

**Published:** 2023-03-16

**Authors:** Daniela M. Zolezzi, Norberto E. Naal-Ruiz, Luz María Alonso-Valerdi, David I. Ibarra-Zarate

**Affiliations:** a Escuela de Ingeniería y Ciencias, Tecnológico de Monterrey, Monterrey 64849, Nuevo León, México; b Escuela de Ingeniería y Ciencias, Tecnológico de Monterrey, Vía Atlixcáyotl 2301, Puebla 72453, México; c Center for Neuroplasticity and Pain, Department of Health Science and Technology, Aalborg University, Aalborg 9220, Denmark

**Keywords:** EEG raw data, EEG resting state, chronic neuropathic pain, pain classification, Brief Pain Inventory, painDETECT, pain severity, pain scores

## Abstract

Thirty-six chronic neuropathic pain patients (8 men and 28 women) of Mexican nationality with a mean age of 44±13.98 were recruited for EEG signal recording in eyes open and eyes closed resting state condition. Each condition was recorded for 5 min, with a total recording session time of 10 min. An ID number was given to each patient after signing up for the study, with which they answered the painDETECT questionnaire as a screening process for neuropathic pain alongside their clinical history. The day of the recording, the patients answered the Brief Pain Inventory, as an evaluation questionnaire for the interference of the pain with their daily life. Twenty-two EEG channels positioned in accordance with the 10/20 international system were registered with Smarting mBrain device. EEG signals were sampled at 250 Hz with a bandwidth between 0.1 and 100 Hz. The article provides two types of data: (1) raw EEG data in resting state and (2) the report of patients for two validated pain questionnaires. The data described in this article can be used for classifier algorithms considering stratifying chronic neuropathic pain patients with EEG data alongside their pain scores. In sum, this data is of extreme relevance for the pain field, where researchers have been seeking to integrate the pain experience with objective physiological data, such as the EEG.

**Specifications Table**

**Table:** 

Subject	Biological Sciences: Neuroscience
Specific subject area	Neuroscience: Sensory Systems, Electrophysiology
Type of data	Electrophysiological recordings – signals recorded through electroencephalography Text files – electrode positions Excel file – ID with demographics, clinical history, and questionnaire results
How the data were acquired	Electroencephalographic recordings 24-channel EASYCAP electrode cap by the 10/20 international system SMARTING EEG Bluetooth amplifier Sampling frequency: 250 Hz Resolution: 24 bits Bandwidth: 0.1 – 100 Hz Openvibe for signal acquisition Electrode impedances were kept below 5 kΩ. Right (M2) and left mastoid (M1) electrodes as offline reference Questionnaires painDETECT and Brief Pain Inventory were used in their Spanish validated version. The English version is uploaded in the repository
Data format	Questionnaire results and demographics– excel Electrophysiological signals – raw Electrode positions – text
Description of data collection	All patients had: age above 18 years old, chronic neuropathic pain for more than 3 months, long-term pharmacological treatment for at least 4 weeks prior to the EEG recording, absence of a major psychiatric disorder (i.e., schizophrenia, major depressive disorder, bipolar disorder), absence of a neurological disorder (i.e., epilepsy, tinnitus), and Total Score >12 points of painDETECT(the questionnaire outcome was confirmed by the clinical history of the patient).
Data source location	• Institution: Tecnológico de Monterrey • City/Town/Region: Monterrey, Nuevo León • Country: México • Latitude and longitude: 25.647903503278236,−100.28928530251193
Data accessibility	*Electrophysiological recordings, text file and excel file are available in a public repository.* Repository name: Mendeley Data Data identification number: doi:10.17632/yj52xrfgtz.4 Direct URL to data: https://data.mendeley.com/datasets/yj52xrfgtz/4 Version 4 *M. Zolezzi, Daniela; Naal-Ruiz, Norberto E.; Alonso Valerdi, Luz María; Ibarra Zárate, David Isaac (2023), “Chronic Neuropathic Pain: EEG data in eyes open (5 min) and eyes closed (5 min) with questionnaire reports”, Mendeley Data, V4*, doi:10.17632/yj52xrfgtz.4
Related research article	D.M. Zolezzi, L.M. Alonso-Valerdi, D.I. Ibarra-Zarate, EEG frequency band analysis in chronic neuropathic pain: A linear and nonlinear approach to classify pain severity, Comput Methods Programs Biomed. 230 (2023) 107349. https://doi.org/10.1016/j.cmpb.2023.107349.

## Value of the Data

• To our knowledge, this is the first available dataset containing the electroencephalographic activity of chronic neuropathic pain patients alongside the patient's pain reports from two validated pain questionnaires. This information is useful for characterization and classification for better pain management.
• Pain researchers, neuroscientists, data scientists, or clinicians would benefit from exploring different pain characterization and stratification methods.
• This data can be reused to understand the relation of electrophysiological data, such as neuronal brain oscillations, with the patient's experience of pain. For example, creating new algorithms for online monitor systems that assess pain through objective measures. Also, it could be used to train supervised and unsupervised classifiers with different psychological factors that are assessed through the questionnaires.

## 1 Objective

Chronic neuropathic pain (NP) is a challenge for clinicians and patients. It is present in about 7-10% of the general population in adults [1] and up to 6% of infants [2]. Chronic NP is a direct consequence of a lesion or disease affecting the nervous system that is persistent or recurrent for more than 3 months [3]. Maladaptive changes in the nervous system allow for unbearable pain described as stabbing, throbbing, tingling,or electrical [4]. Despite increased pharmacological trials for chronic NP, efficacy has failed due to a lack of proper characterization and stratification. The electroencephalogram (EEG) offers advantages as a non-invasive assessment tool with a lower cost and simpler methodology than other imaging techniques [5]. However, much research is still needed to develop methods for reliable pain characterization using EEG. Thus, this dataset which contains physiological (i.e., brain activity) and psychological (i.e., questionnaire reports), complements the collective approach of the pain field to improve the assessment and management of chronic NP. This data article adds value to the original research article by allowing researchers to explore the data with other linear and nonlinear methods as proposed in [6].

## 2 Data Description

This section describes the data in the Mendeley Data repository.

### 2.1 Electrophysiological recordings

**Fig. 1:**
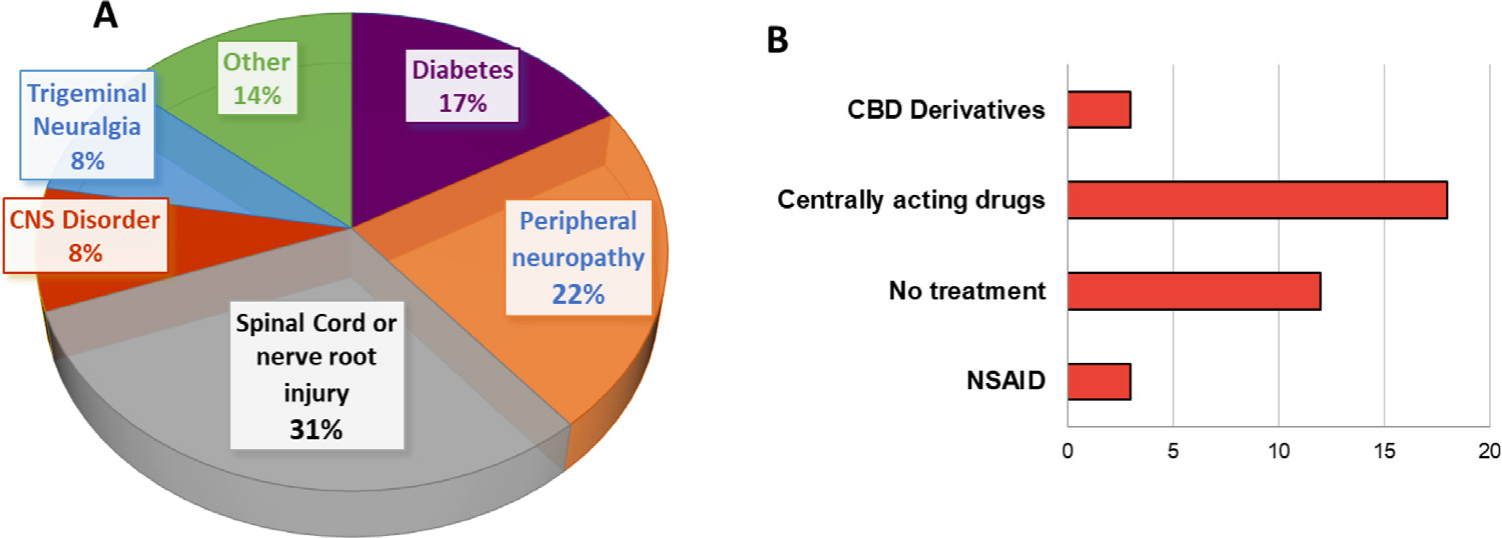
The etiologies of the NP sample were spinal cord injury (31%), painful peripheral neuropathy (23%), diabetes (17%), trigeminal neuralgia (8%), CNS disorder (8%), and other (14%). The type and frequency of the pharmacological treatment were: eighteen patients (n=18) taking centrally acting drugs for over a year, twelve patients (n=12) were not taking medication, three patients (n=3) were on cannabidiol derivatives (CBD), and three (n=3) took nonsteroidal anti-inflammatory (NSAID) drugs for pain attacks.

The raw EEG data is provided for every patient ID in .gdf format. A General Data Format (GDF) file can be read and written by BioSig, an open-source software library for biomedical signal processing implemented in Octave/Matlab, C/C++, Python, Java and R [7,8]. The open-source software for opening GDF files can be downloaded in [7], and detailed information on GDF file type can be found in [8]. Electrophysiological signals correspond to the spontaneous activity of 36 chronic NP patients at resting state for 10 min in two conditions: eyes open (first 5 min) and eyes closed (last 5 min). The datasets contain an extra five seconds at the beginning of each recording, it is recommended to use the signals starting from the second five. The causes for NP in the studied sample and the pharmacological treatment of the studied population are described in Fig. 1. Other details concerning their clinical history and area of pain can be revised in the Excel file.

### 2.2 Excel file

The excel file contains three sheets. The first contains the demographics of each patient: age, gender, etiology, and clinical history. The second and third sheets contain the answers to the pain questionnaires, Brief Pain Inventory (BPI) and painDETECT questionnaire (PDQ), which were used in their validated Spanish version [9,10]. The answers have been translated according to their English version [11,12] and can be found in the data repository. An overview of the questionnaire results is described in the Supplementary Material of [6].

#### 2.2.1 Brief Pain Inventory answers

The second sheet contains the answers to the BPI. The *functionality* category of the BPI is divided into seven items: general activity, mood, ability to walk, normal work, relationships with other people, rest, and enjoyment of life. Each item is measured on an 11-point scale from 0 to 10, where 0 is no pain or no interference, and 10 is worst pain or complete interference.

#### 2.2.2 painDETECT answers

The third sheet contains the answers to the nine items of the PDQ. Two questions had to be acquired differently for dataset purposes since they required for patients to *draw* and *mark*. When the questionnaire asks patients to “Please *mark* your main area of pain”, we asked them instead to *write* the main area of pain as specifically as possible. Also, for the question “If yes, please *draw* the direction in which the pain radiates”, we asked them to *write* the direction in which it irradiates and to be as explicit as possible (e.g., from the right arm to the head, from the left leg towards the back, etc.). For the PDQ, a total score between -1 and 38 can be calculated from the nine items, with a greater probability of having NP with higher scores. A score less than or equal to 12 indicates pain with a modest probability of being NP, greater than or equal to 19 means that there is a 90% probability that NP exists. The final score of each patient can be calculated with the uploaded questionnaire in the repository.

### 2.3 Text files

Two .txt files of channel locations (mBrain 24 ch locations and rereferenced 22 ch, without M1 and M2) are available. The rereferenced 22 ch text file can be used when preprocessing the data after referencing to M1 and M2.

## 3 Experimental Design, Materials and Methods

### 3.1 Sample size calculation

The sample size was calculated in line with Eq. (1), with a two-tailed Z distribution to evaluate the similarity between a control group (null hypothesis) and the NP group (alternate hypothesis). Calculations were performed in MATLAB R2020a (The Mathworks, Inc., Natick, MA, USA) with the Statistics and Machine Learning Toolbox [13].

(1) $$Power=\phi \;\left[z_{\propto }+\frac{\left(\mu_{0}-\mu_{1}\right)*\sqrt{n}]}{\sigma }\right]$$

Note that for Eq. (1), the power depends on four factors: ∝, $|\mu_{0}+\mu_{1}$|, σ, and *n.* Thus, to compute the power with this formula, an expected value from the control group ($\mu_{0}\;\text{with}\;\text{its}\;\text{variance}\;\sigma )$ and from the NP group ($\mu_{1}$) is necessary. To solve for *n,* see Eq. (2), where 1-ß is the power and ∝, is the significance level:

(2) $$n=\frac{{\left(\;z_{1-\propto /2}+\;z_{1-ß}\right)}^{2}\sigma^{2}}{\;{\left(\mu_{0}-\mu_{1}\right)}^{2}\;}$$

The parameter used to estimate significant differences between the groups was the individual alpha frequency (IAF) value, an EEG biomarker taken from the analysis of the alpha band, which is modulated by lifestyle, diet, exercise, sex, age, and other factors. Moreover, it can be affected by neuropathologies such as tinnitus and NP due to the mechanism of thalamocortical dysrhythmia. It has been reported that the IAF of tinnitus and NP patientsis equivalent, given the same thalamocortical dysrhythmia as the background neuronal activity [14]. Thus, to approximate the unknown value of the IAF of NP patients, EEG data from tinnitus patients and the control group was requested from the authors of a previous study [15]. For the control group, a mean of 10 Hz and a standard deviation of 1 Hz were obtained from 18 healthy subjects (11 men and 7 women, with a mean age of 38+-10.2). These results support those found in another group of healthy patients from ages 20-80 years reported in [16] and in [17]. On this basis, $\mu_{0}$ (the mean of the null hypothesis) was set to 10 Hz, and the σ to 1 Hz. For patients with tinnitus, the resulting IAF was calculated from 65 patients with tinnitus (40 women and 25 men; mean age 54.92+-11.79) and resulted in 9.5 Hz ($\mu_{1}$, mean of the alternate hypothesis), which is also supported by the results of another NP study [18]. Considering a power between 0.8 and 0.9, the following parameters were applied in Eq. (2), $\mu_{0}=10$,$\;\mu_{1}=9.5,\;\propto =0.05,\;and\;\sigma =1$. According to Eq. (2), for a power of 0.8, ß=0.2, and ∝= 0.05, n=32, meaning that 32 NP patients are needed to reach significant differences between NP and a control group. For a power of 0.9, ß=0.1 and ∝= 0.05, n=43. Thus, the objective was to recruit 32 to 43 patients with chronic NP.

### 3.2 Questionnaire election

As stated previously, two validated questionnaires in Spanish [[9], [10]] were used to characterize NP [19]. The reasons for selecting these questionnaires were the following:

1. The PDQ [12] is an NP diagnostic tool and was used as an inclusion criterion. Additionally, this questionnaire specifies four different patterns of pain fluctuation (see attached questionnaire). These patterns can be important information for subsequent analysis of EEG signals. In addition, pain qualities are used with various adjectives that can help the patient respond according to his or her symptoms (e.g., burning, tingling, sudden pains, electric shocks, numbness).
2. The BPI isan NP assessment tool [11]. This second questionnaire was chosen because the patient reports the impact that NP causes in his or her daily life. For example, the patient responds the degree of pain relief relative to the individual treatment and the effect of pain on work, mood, ability to walk, or relationships.

### 3.3 Justification for allowing patients to have medication

For ethical reasons, patients were allowed to continue their medication if they had been receiving it at least 4 weeks before the study, which is supported by a prior EEG study where patients received co-analgesic treatment (anticonvulsants and antidepressants) [20]. Moreover, the sub-chronic dose (25 mg for more than 15 days) of amitriptyline (an antidepressant given for NP) did not affect the P3 component (EEG evoked potential) in patients with NP [21]. Finally, no significant differences were found between NP patients with or without central drugs (opiates and antidepressants) in EEG studies at resting state andat a bandwidth of 2-18 Hz [22].

### 3.4 EEG recording equipment

**Fig. 2:**
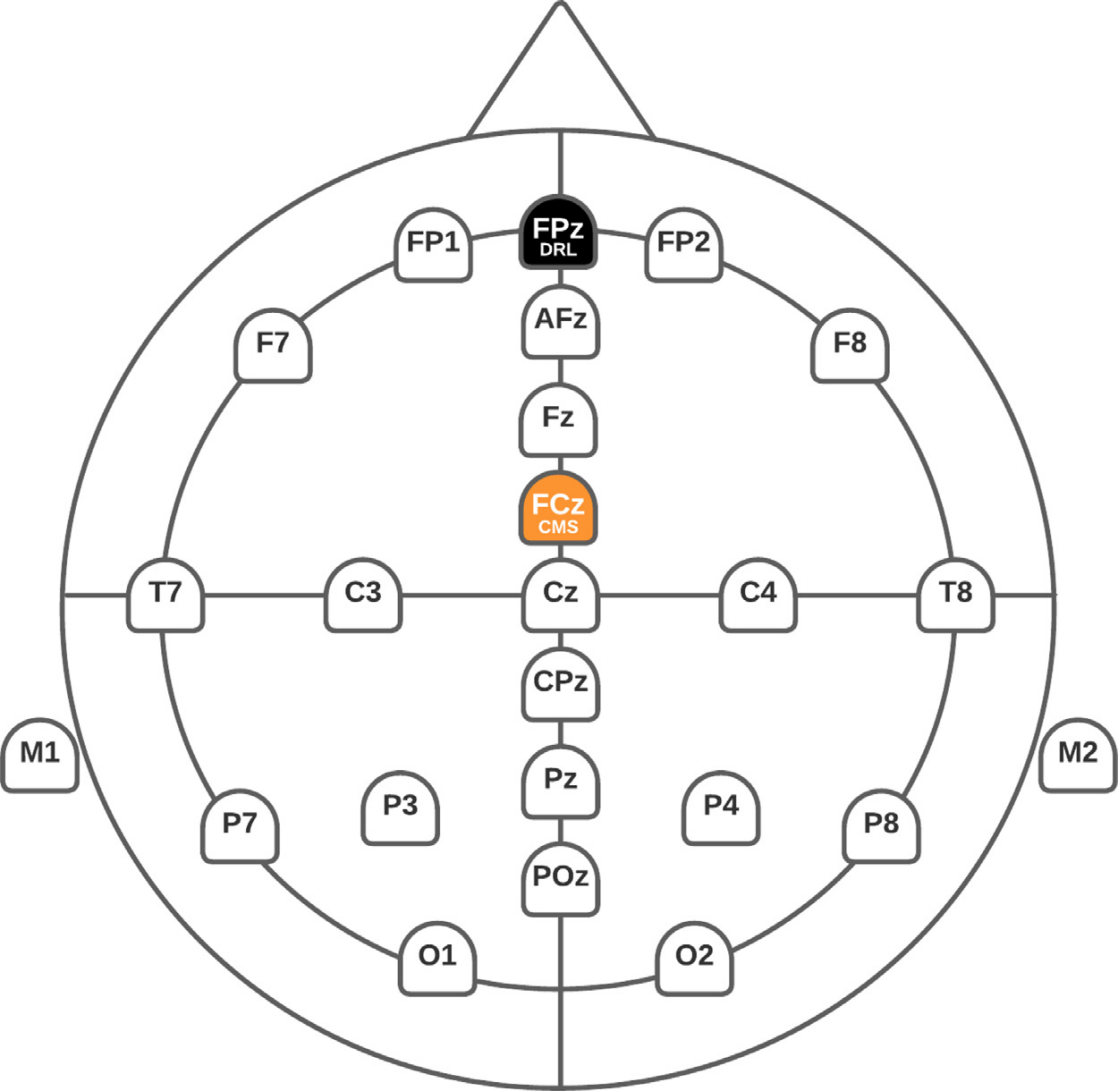
Topographic map for the 24 channels positioned according to the 10/20 system for the Smarting mBrain cap. M1 and M;2 correspond to the earlobe (i.e., mastoid) electrodes for offline reference.

**Fig. 3:**
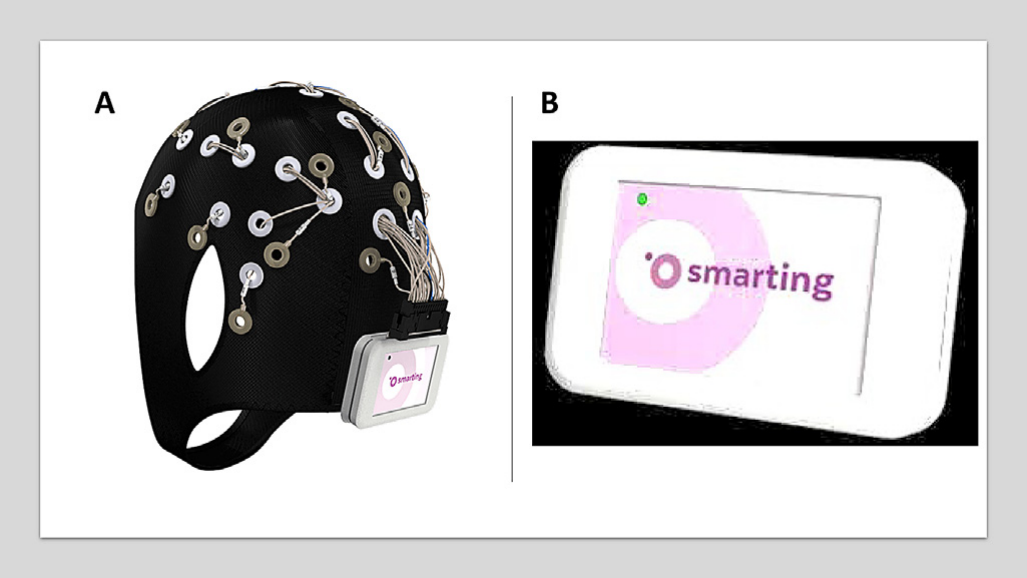
Laboratory Resources of the Neuroengineering and Neuroacoustics Research Group. (A) MBrainTrain cap with 24 Ag/AgCl electrodes. (B) Smarting device used for communication between electrodes and recording device by means of Bluethooth.

Ten minutes of spontaneous EEG data were recorded using 22 electrodes (Fp1, Fp2, AFz, F7, F3, Fz, F4, F8, T7, C3, Cz, C4, T8, CPz, P7, P3, Pz, P4, P8, POz, O1, O2) positioned according to the International 10/20 System with two earlobe references (M1 and M2). The channel positions for the data are shown in Fig. 2, which has also been uploaded to the dataset files. Fig. 3A and B shows the Smarting mBrain cap and amplifier used. The input impedance of the system is 1 GΩ. The input referred noise is less than 1 µV. It has a resolution of 24 bits. The sampling frequency used was 250Hz. The bandwidth is 0-250 Hz, with a flat frequency response of 0-133 Hz. The communication type is wireless Bluetooth v2.1 (See Fig. 3B). OpenViBe software [24] was used to implement the experimental paradigms and record the EEG signals. The sampling frequency was 250 Hz, and the bandwidth was between 0.1 and 100 Hz. Electrode impedances were kept below 5 kΩ.

### 3.5 Experimental procedure

**Fig. 4:**
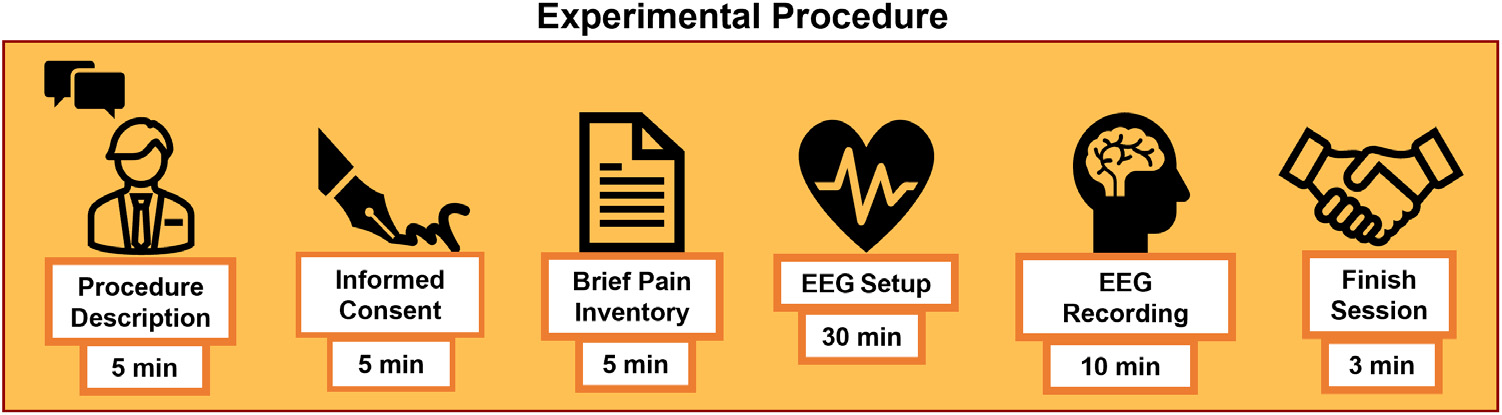
Experimental Procedure for the Recording Session. First, the procedure was explained to the patient thoroughly. Second, the informed consent was signed. Third, the BPI questionnaire was answered. Fourth, the EEG equipment was installed. Finally, the spontaneous EEG recording paradigm started and the session ended in approximately 55 min.

The experimental procedure was as follows [6,23]. First, the recording procedure was described to each patient. If the patient agreed to participate, they signed the informed consent. Afterward, patients answered the BPI questionnaire, and the EEG was installed. For the EEG recording, patients sat in an upright position. For the first five minutes, they were asked to keep their eyes opened and fixed on a white cross in a dark background of a monitor 50 cm away. At the end of the first 5 minutes, the cross disappeared, and patients closed their eyes for the last 5 minutes until an auditory beep marked the end of the recording. A simplified diagram of the experimental procedure is depicted in Fig. 4.

## Ethics Statements

Prior to the start of the experiment, all patients provided written informed consent according to the World Association Declaration of Helsinki. This study was approved by the Clinical Investigation Ethics Committee of Tecnológico de Monterrey (number: P000369-DN-RespElectro-CI-CR005).

## CRediT Author Statement

**Daniela M. Zolezzi:** Literature Research, Data Recording, Writing – original draft; **Norberto E. Naal-Ruiz:** Data Recording, Supervision; **Luz María Alonso-Valerdi:** Supervision, Conceptualization – review & editing; **David I. Ibarra-Zarate:** Data Supervision, Writing – review & editing.

## Declaration of Competing Interest

The authors declare that they have no known competing financial interests or personal relationships which have or could be perceived to have influenced the work reported in this article.

## Data Availability

Chronic Neuropathic Pain: EEG data in eyes open and eyes closed with questionnaire reports (Original data) (Mendeley Data). Chronic Neuropathic Pain: EEG data in eyes open (5 min) and eyes closed (5 min) with questionnaire reports (Original data) (Mendeley Data).
